# Disseminated *Candida lusitaniae*: Nosocomial Acquisition Secondary to an Indwelling Urinary Catheter

**DOI:** 10.1155/2021/6632730

**Published:** 2021-06-17

**Authors:** Ali Raja, Julia Park

**Affiliations:** ^1^Mountain Vista Medical Center, Mesa, AZ, USA; ^2^Midwestern University, Glendale, AZ, USA

## Abstract

*Candida lusitaniae* is a rare opportunistic pathogen, and its most common risk factors include immunocompromised patients often with an underlying malignancy. It commonly displays resistance to amphotericin B, and historically, echinocandins have been considered first-line treatment. We present a 77-year-old male with a history of diabetes mellitus. He was treated for cellulitis and discharged to a skilled nursing facility with an indwelling urinary catheter. Despite recommendations from the medicine team to remove the catheter, the patient refused even after discussing the risks and benefits. He returned to the hospital 3 weeks later with symptoms of dysarthria, right-sided facial droop, and right-sided weakness. Ultimately, he was determined to have fungemia and native valve endocarditis due to *Candida lusitaniae* stemming from his indwelling urinary catheter. He was treated with micafungin, but repeated blood cultures continued to grow *C. lusitaniae*, and he eventually expired following withdrawal of care. We present this case report to illustrate a rare occurence of *Candida lusitaniae* in a patient without typical risk factors. *C. lusitaniae* fungemia is an extremely uncommon disease in patients without underlying malignancy. Despite this lack of apparent, classic risk factors, our patient developed endocarditis of his native valve due to *C. lusitaniae* fungemia from an indwelling urinary catheter. The ability of this organism to form biofilms, and its rapid mutation rate, makes treating *C. lusitaniae* very difficult. The treatment of choice for *C. lusitaniae* endocarditis is surgical intervention due to biofilm formation on the cardiac valves. Medical treatment recommendations are currently fluconazole, which is in contrast to the historical use of echinocandins. Infection due to *Candida lusitaniae*, though rare, should be remembered by clinicians. This particular fungal agent is especially difficult to treat due to its multiple virulence factors. Additionally, the use of indwelling urinary catheters should only occur when proper indications are present and should be promptly discontinued when their placement is no longer necessary.

## 1. Introduction

*Candida lusitaniae* fungemia is a very rare disease that affects patients with underlying malignancy and/or undergoing chemotherapy. Fungemia with endocarditis of native valves due to *C. lusitaniae* is an even more unique case, which necessitates discussion. This case report discusses an interesting occurrence of *C. lusitaniae* fungemia and endocarditis in a patient without underlying malignancy, all originating from an indwelling urinary catheter. A literature review is then discussed, including a review on this pathogen's multiple virulence factors and the best initial approach to treat it.

## 2. Case Presentation

A 77-year-old Caucasian male with a past medical history of insulin-dependent diabetes mellitus, dyslipidemia, hypertension, and chronic kidney disease stage IIIa presented to the emergency department due to bilateral lower extremity pain. The patient was diagnosed and treated for lower extremity cellulitis. Due to pain of his lower extremities with ambulation, the patient requested an indwelling urinary catheter. Therefore, urinary catheter placement was performed on the patient's request. Prior to discharge to the skilled nursing facility, discontinuation of the indwelling urinary catheter was recommended multiple times to the patient, but he refused the catheter's removal. He was discharged to the skilled nursing facility after having completed his antibiotic course. He was to continue furosemide, gabapentin, and also nystatin cream for prophylactic prevention of fungal infection on his feet bilaterally.

Nearly three weeks after discharge to the skilled nursing facility, the patient was brought to the emergency department by emergency medical services due to signs of a possible stroke observed at the facility. He was demonstrating dysarthria, right-sided facial droop, and right-sided weakness. These symptoms were first noted about 2.5 hours prior to arrival. Computed tomography head showed no acute abnormality. The patient was not a candidate for alteplase due to an elevated INR.

Initial emergency department vitals showed a temperature of 38.3. After blood cultures were drawn, a single dose of one gram of ceftriaxone was given to the patient. Later that day, the patient's antibiotics were broadened to vancomycin and cefepime. On his physical exam, the patient's speech remained dysarthric, and he also exhibited a right-sided facial droop with right-sided weakness. The indwelling urinary catheter from his previous admission was still in place. His labs were remarkable for a creatinine level of 3.50, a blood urea nitrogen of 105, a potassium level of 5.2, and a serum bicarbonate of 17. His white blood cell count was within normal limits. Computed tomography angiography of the head and neck vessels was performed in the emergency department even with his acute on chronic kidney injury with a creatinine of 3.50. The computed tomography scans revealed a 50% to 70% stenosis of the left carotid artery and a less than 50% stenosis of the right carotid artery at the bifurcation. The patient underwent magnetic resonance imaging of the brain the following day, which revealed no signs of an acute stroke.

Upon returning to his hospital room after his magnetic resonance imaging scan, the patient was observed to be unresponsive with no palpable pulses. Cardiopulmonary resuscitation was started. Once return of spontaneous circulation was achieved, the follow-up electrocardiogram did not reveal any ST-segment or T-wave changes concerning for ischemia or infarction. Transthoracic echocardiogram with a bubble study revealed an ejection fraction estimated at 50% to 55% without any wall motion abnormalities and a negative bubble study. Alarmingly, the echocardiogram reported “a possible vegetation attached to the tricuspid valve with a separate possible vegetation attached to the Eustachian valve.” It also reported mild-to-moderate tricuspid regurgitation.

At this time, the patient's blood cultures returned positive for fungal growth. Urine cultures were drawn following the blood culture results, and those cultures also demonstrated fungal growth. The patient's blood and urine cultures were finalized as *Candida lusitaniae*, and the patient continued on micafungin 150 mg daily. As this organism is highly uncommon, multiple blood cultures were drawn. However, on 3 different days, the blood cultures resulted positive for *C. lusitaniae*.

As the days progressed, the patient displayed findings consistent with an anoxic brain injury. Therefore, the decision was made with the family to withdraw care, and the patient expired soon after.

## 3. Discussion

The dangers of indwelling urinary catheters without proper care are fairly well known [1]. They lead to around 80% of nosocomial urinary tract infections [2]. Proper training in indwelling urinary catheter insertion and care has been shown to reduce the number of catheter-associated urinary tract infections [1]. An indwelling urinary catheter should be maintained no more than 30 days at a time, and with the European Association of Urology Nurses (EAUN) recommending, no more than 14 days [3]. We postulate that our patient's fungemia was due to his indwelling urinary catheter.

*C. lusitaniae* is a rare opportunistic infection that causes disseminated infections in immunocompromised patients with underlying malignancy and/or undergoing chemotherapy [2, 4, 5]. Our patient does not have the typical risk factors for *C. lusitaniae*. *C. lusitaniae* has been described to contribute to colonization and infection in patients with prolonged hospitalizations and use of broad-spectrum antibiotics [6].

Another unique attribute of *C. lusitaniae* is that many isolates have demonstrated resistance to amphotericin B [4, 7, 8]. *C. lusitaniae* develops antifungal resistance rapidly. It is hypothesized that this ability may be enhanced when biofilm formation occurs [9]. Although biofilm formation by *C. lusitaniae* has been previously documented to occur on central venous catheters [10], we theorize that a biofilm may have formed on our patient's indwelling urinary catheter as our patient never had a central venous catheter.

We are presuming that our patient developed endocarditis due to *C. lusitaniae* as there were transthoracic echocardiogram image findings of vegetations on the tricuspid and eustachian valves. Additionally, the only cultures that were positive were for *C. lusitaniae* on multiple days. As the antibiotics were given after the blood cultures were drawn in the emergency department, we are safely assuming that the culture results are the cause of the echocardiogram findings. We did not pursue a postmortem autopsy due to the family's preferences and wishes. Although we do not have pathology results indicating these findings, we have strong reasons to believe that the culture results were pathologic as they were isolated from two different sites on 3 separate days.

If this patient had survived, the treatment of choice for his *C. lusitaniae* endocarditis is surgery [7, 11]. This is because it is assumed that *C. lusitaniae* forms biofilms on the cardiac valves as well, thus making it difficult to treat without surgical intervention [7].

Given this infectious agent's ability to form biofilms as well as the ability to mutate rapidly to develop resistance to various antifungals makes treating *C. lusitaniae* very difficult. A number of case reports describe successful eradication of *C. lusitaniae* using fluconazole [12–16]. This is in contrast to the historical use of echinocandins and should be taken into consideration in the setting of a *C. lusitaniae* infection.

## 4. Conclusions

Fungemia and endocarditis due to *C. lusitaniae* is incredibly rare and even more so when involving native cardiac valves. Nonetheless, the dangers of less-common pathogens should be considered when making treatment decisions. Our case showed a patient with nontypical risk factors for *C. lusitaniae* infection. Our case also sheds light on the dangers of indwelling urinary catheters without proper care, and this needs to be remembered by all those providing patient care. Finally, based on our experience and from the review of the existing literature, echinocandins may not be the best first option for *C. lusitaniae*, and when this particular fungus is suspected, extra care should be taken when initiating antifungal therapy.

## Data Availability

The health record data used to support the findings of this case report are restricted in order to protect patient privacy. Where appropriate, certain health record data are included verbatim within the article. This case report also provides a discussion of *Candida lusitaniae*. The data used in the discussion were found within peer-reviewed journals and previously published case reports. Appropriate citations and references are included within the article.

## References

[1] Barry J. L., Wenthold R. (2016). Reducing catheter associated urinary tract infections through foley insertion competency assessment upon hire. *American Journal of Infection Control*.

[2] Minari A., Hachem R., Raad I. (2001). *Candida lusitaniae*: a cause of breakthrough fungemia in cancer patients. *Clinical Infectious Diseases*.

[3] Newman D. K. (2007). The indwelling urinary catheter. *Journal of Wound, Ostomy & Continence Nursing*.

[4] Hawkins J. L., Baddour L. M. (2003). *Candida lusitaniae* infections in the era of fluconazole availability. *Clinical Infectious Diseases*.

[5] Mishra R., Kelly P., Toolsie O., Ayyadurai P., Adrish M. (2017). Uncommon cause of fungemia in a patient with renal cell cancer. *Medicine*.

[6] Sanchez V., Vazquez J. A., Barth-Jones D., Dembry L., Sobel J. D., Zervos M. J. (1992). Epidemiology of nosocomial acquisition of *Candida lusitaniae*. *Journal of Clinical Microbiology*.

[7] Michel R. G., Kinasewitz G. T., Drevets D. A., Levin J. H., Warden D. W. (2009). Prosthetic valve endocarditis caused by *Candida lusitaniae*, an uncommon pathogen: a case report. *Journal of Medical Case Reports*.

[8] Rahmati E., Correa A. J., She R. C. (2020). A budding case of infectious endocarditis: *Candida lusitaniae*. *ID Cases*.

[9] Demers E. G., Biermann A. R., Masonjones S. (20181). Evolution of drug resistance in an antifungal-naive chronic *Candida lusitaniae* infection. *Proceedings of the National Academy of Sciences*.

[10] Simitsopoulou M., Kyrpitzi D., Velegraki A., Walsh T. J., Roilides E. (2014). Caspofungin at catheter lock concentrations eradicates mature biofilms of *Candida lusitaniae* and *Candida guilliermondii*. *Antimicrobial Agents and Chemotherapy*.

[11] Patel A., Almuti W., Polenakovik H. (2014). Acute limb ischemia due to *Candida lusitaniae* aortic valve endocarditis. *Heart & Lung*.

[12] Crum-Cianflone N. F., Butera M. (2007). *Candida lusitaniae* meningitis. *Infectious Diseases in Clinical Practice*.

[13] Pietrucha-Dilanchian P., Lewis R. E., Ahmad H., Lechin A. E. (2001). *Candida lusitaniae* catheter-related sepsis. *Annals of Pharmacotherapy*.

[14] Tikotekar A., Naik A., Fisher B., Rahman H., Lopez R. (2008). *Candida lusitaniae*: an emerging cause of pleuropulmonary infection. *Chest*.

[15] Jeragh A., Ahmad S., Naseem J., Khan Z. U. (2007). *Candida lusitaniae* arthritis in an intravenous drug user. *Mycoses*.

[16] Wawrysiuk S., Rechberger T., Futyma K., Miotła P. (2018). *Candida lusitaniae*—a case report of an intraperitoneal infection. *Menopausal Review*.

